# An Epidemiological Survey of Intestinal Parasitic Infection and the Socioeconomic Status of the Ethnic Minority People of Moken and Orang Laut

**DOI:** 10.3390/tropicalmed8030161

**Published:** 2023-03-06

**Authors:** Suphaluck Wattano, Kamonwan Kerdpunya, Phongton Keawphanuk, Saowalak Hunnangkul, Sumas Loimak, Aunchalee Tungtrongchitra, Metta Wongkamchai, Sirichit Wongkamchai

**Affiliations:** 1 Department of Parasitology, Faculty of Medicine Siriraj Hospital, Mahidol University, Bangkok 10700, Thailand; 2 Koh Lipe Health Promoting Hospital, Satun 91000, Thailand; 3 Department of Research and Development, Faculty of Medicine Siriraj Hospital, Mahidol University, Bangkok 10700, Thailand; 4 Filaria Project, Phikulthong Royal Development Study Center, Narathiwat 96000, Thailand

**Keywords:** Moken, Orang Laut, neglected tropical diseases, NTDs, intestinal parasitic infection, IPIs, helminth, protozoa

## Abstract

Ethnic minority groups are often subjected to exclusion, social and healthcare marginalization, and poverty. There appears to be important linkages between ethnic minority groups, poor socioeconomic status, and a high prevalence of parasitic infection. Data regarding the prevalence and health effects of IPIs are necessary in the development and implementation of targeted prevention and control strategies to eradicate intestinal parasitic infection in the high-risk population. Thus, we investigated for the first time the intestinal parasitic infection status (IPIs), the socioeconomic status, and sanitary condition in the communities of Moken and Orang Laut, the ethnic minority peoples living on the coast of southwest Thailand. A total of 691 participants participated in the present study. The information concerning socioeconomic status and sanitary condition of the study population was obtained by personal interviews using a picture questionnaire. Stool samples were collected and examined for intestinal parasitic infection using direct wet smear and formalin-ethyl acetate concentration techniques. The results revealed that 62% of the study population were infected with one or more types of intestinal parasites. The highest prevalence of intestinal parasitic infections was found in the 11–20-year-old age range group. A statistically significant difference of IPIs among the three communities were observed (*p* < 0.0001). There was a statistical difference concerning 44 multiple infections of soil-transmitted helminths (STHs) (*p* < 0.001), whereas no statistically significant difference in multiple infections of protozoa was observed (*p* > 0.55). The results also displayed the significant difference in socioeconomic status and sanitary condition among the Moken living in Ranong and Phang Nga and the Orang Laut living in the Satun province (*p* < 0.001). Our study found no direct association between parasitic infection status and ethnic/geographic features; however, socioeconomic status is the key factor associated with prevalence of intestinal parasitic infection, with the observation that the higher prevalence of IPIs is due to a low socioeconomic status, consequently leading to poor hygiene and sanitation practices. The picture questionnaire played a major role in information gathering, especially from those of low or no education. Lastly, data pertaining to the species of the parasites and the mode of transmission assisted in the identification of group-specific vulnerabilities and shortcomings that can be utilized in education and corrected to reduce the prevalence of infection in the study areas.

## 1. Introduction

Neglected tropical diseases (NTDs) comprise a group of diseases, including infections caused by parasites in tropical and subtropical regions of the world, especially among marginalized populations facing poverty. NTDs affect more than one billion people globally [1]. Intestinal parasitic infections (IPIs) are considered to be one of the NTDs that remain an important public health problem in low- and middle-income countries in the tropical and subtropical zones of the world [2,3,4]. Protozoans and soil-transmitted helminths (STHs) are the two main etiologies of IPIs in humans [5,6,7]. Globally, nearly 3.5 billion people are infected with IPIs [8], and the highest prevalence of IPIs reported in recent decades was in southeast Asian countries (SEA) [7,9,10]. A national survey conducted in 2009 reported that the prevalence of helminthiasis was 18.1% in the Thai population [11]. According to the World Health Organization, preschool children, school-age children, adolescents, and women of reproductive age are at greater risk of STH infection. STH infections are still considered a significant health problem in rural and underdeveloped areas of Thailand [12,13].

Intestinal helminth infections may cause adverse effects on health, such as malnutrition, anemia, and impaired growth and cognitive development [14,15,16,17]. IPIs in pregnant women may increase inadequate weight gain, intra-uterine growth retardation, and a low birth weight in newborn babies [18]. Additionally, they may contribute substantially to impaired mental and educational development in children [14,15,16,19]. Protozoa infections are a major contributor to malnutrition and may contribute to changes in the composition of the microbiota and activation of immune responses [6,20]. *Entamoeba histolytica* causes acute diarrhea while *Giardia intestinalis* causes chronic diarrhea, both of which can lead to post-infectious syndrome [21]. Repetitive parasitic infections can lead to adverse consequences that can be passed on from one generation to the next, creating a vicious cycle of poverty and poor health [22]. Consequently, the necessity to design appropriate and cost-effective strategies for treatment and control of intestinal parasitic infections based on a practical knowledge of epidemiological patterns is of great importance [23].

Baseline surveys at the start of the control program are recommended by the World Health Organization [24,25]. Baseline surveys provide information on the present status in order to estimate the degree of necessity for intervention in the population. It provides essential data and serves as a guide for the development of infection controlling programs at the district, regional, and even national levels. Furthermore, data regarding the prevalence and health effects of IPIs will help in the development and implementation of targeted prevention and control strategies to eradicate intestinal parasitic infection in the high-risk population [26].

Ethnic minority groups are often subjected to exclusion, social and health care marginalization, and poverty. These issues render them vulnerable to otherwise uncomplicated and curable tropical diseases, including parasitic infection. Correlations between ethnic minority groups, low socio-economic status, and high prevalence of tropical diseases, including parasitic infections, are significantly observed. Some crucial factors that play an important role in promoting tropical diseases include lack of health care and a generally poor health status, poverty and social marginalization, as well as low education and literacy [27].

Moken (Mawken or Morgan), a group of Austronesian people of the Mergui Archipelago, and Orang Laut (Urak Lawoi), another ethnic group, are commonly referred to as the seafaring ethnic minority groups living on coastal islands of SEA. These sea nomads were the first, and for a long period, the only inhabitants of these islands. They are Malay originated people, including those of the Mergui Archipelago islands of Myanmar and Thailand. Moken in Thailand are found on the coastal area in southwest regions of Thailand, i.e., Chang, Lao, and Payam islands in Ranong province, and in Ban Thub Tawan village in Phang Nga province. Most Moken spend most of their daily activities on small, covered wooden boats [28].

Orang Laut in Thailand reside on Lipe island in Satun province, or in Rawai and Ban Tukae villages in Phuket province [29]. However, no epidemiological data on IPIs are available concerning these ethnic minority groups.

Studies of IPIs have conducted in the areas of southern Thailand, which focused on adults, the elderly, school-age children, and village health volunteers [11,12,30,31,32,33,34,35], but no study of IPIs has ever been conducted in the ethnic minority peoples. Thus, we focus our interest in the Orang Laut and Moken, the ethnic minority peoples residing on the coast of southwest Thailand.

We conducted the first epidemiology survey on IPIs prevalence and its associate risk factors in two ethnic minority groups, the Moken and Orang Laut, living in the coastal area of southwest Thailand, in order to provide baseline data on IPIs. This will deepen the understanding of any underlying causal factors and will determine their public health and socioeconomic situation.

## 2. Materials and Methods

### 2.1. Study Areas and Study Population

**Figure 1:**
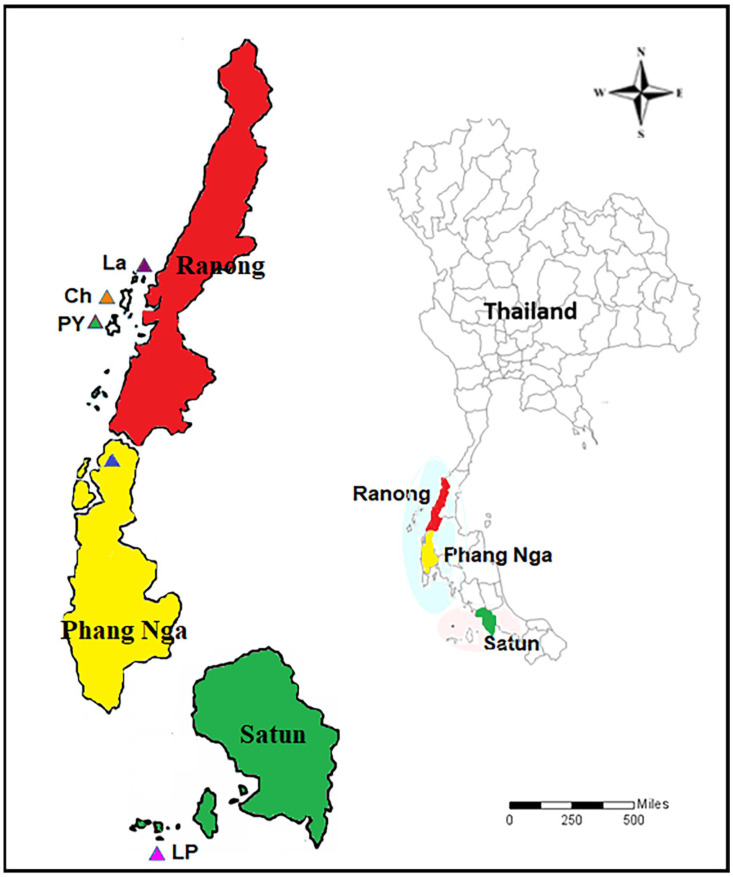
Locations of study areas. Chang Island (CH), Payam Island (PY), Lao Island (LA), Ban Thub Tawan Village (TT), and Lipe Island (LP) as a part of Ranong, Phang Nga, and Satun Provinces, Thailand.

The cross-sectional intestinal parasitic infection survey, as well as an interview to determine the associated factors, was conducted in the ethnic minority groups living in the coastal area of southwest Thailand from October to December 2021. The study population consists of (1) Moken living on Lao Island, Pyam Island, and Chang Island in Ranong province, (2) Moken living in Ban Thup Tawan, the coastal village in Phan Nga province, and (3) Orang Laut living on Lipe island of Satun province (Figure 1).

The sample size was calculated using the infinite population proportion formula Z2 1-α/2 p (1-p)/d2, where the prevalence of intestinal parasites from a previous study [12] (p) was 18.42%. The d2 was 6, and the allowable error and type I error (α) were 0.03 and 0.05, respectively. Calculation yielded a size of 642 subjects. Recruitment was increased by 7% to compensate for reversal in the decision to participate and missing data.

The protocol for this study was approved by the Siriraj Institutional Review Board (SIRB), Faculty of Medicine, Siriraj Hospital, Mahidol University, Bangkok, Thailand (approval no. Si 355/2021). Villagers were informed prior to the study by the village health-volunteers. Verbal consent to participate in the present study was given by the willing inhabitants in each village. For children younger than 11 years old, the parent(s) or guardian(s) of the participant was informed prior to the study. Regarding the low literacy of the study participants, a waiver of written informed consent was approved by the IRB.

The 780 participants from the study areas, i.e., Ranong, Phang Nga, and Satun provinces, Thailand, voluntarily participated in this study. The Moken and Orang Laut teenagers who are studying in Thai schools helped us to communicate with the study participants. The information concerning the socioeconomic status and sanitary condition of the study population was obtained by personal interviews using a picture questionnaire. Each participant was then given a labeled plastic container to collect stool samples after receiving proper instructions.

### 2.2. Sample Collection and Parasitological Diagnosis

To study the prevalence of intestinal parasitic infection (IPIs), one-time stool samples were collected from the study participants. Each stool sample collected was preserved in 10% formalin and transported to the laboratory of the Department of Parasitology, Faculty of Medicine at Siriraj Hospital, Mahidol University, Bangkok, Thailand, for detection of intestinal helminths and protozoa by the direct wet smear method according to the procedures in the WHO guidelines [36]. For all samples that reported as parasite-negative by the direct wet smear, parasite detection was further performed using a formalin-ethyl acetate concentration [36]. The species of parasites found in stool specimens by simple smear or/and formalin-ethyl acetate concentration methods were reported.

### 2.3. Socioeconomic Status and Sanitary Condition

**Figure 2:**
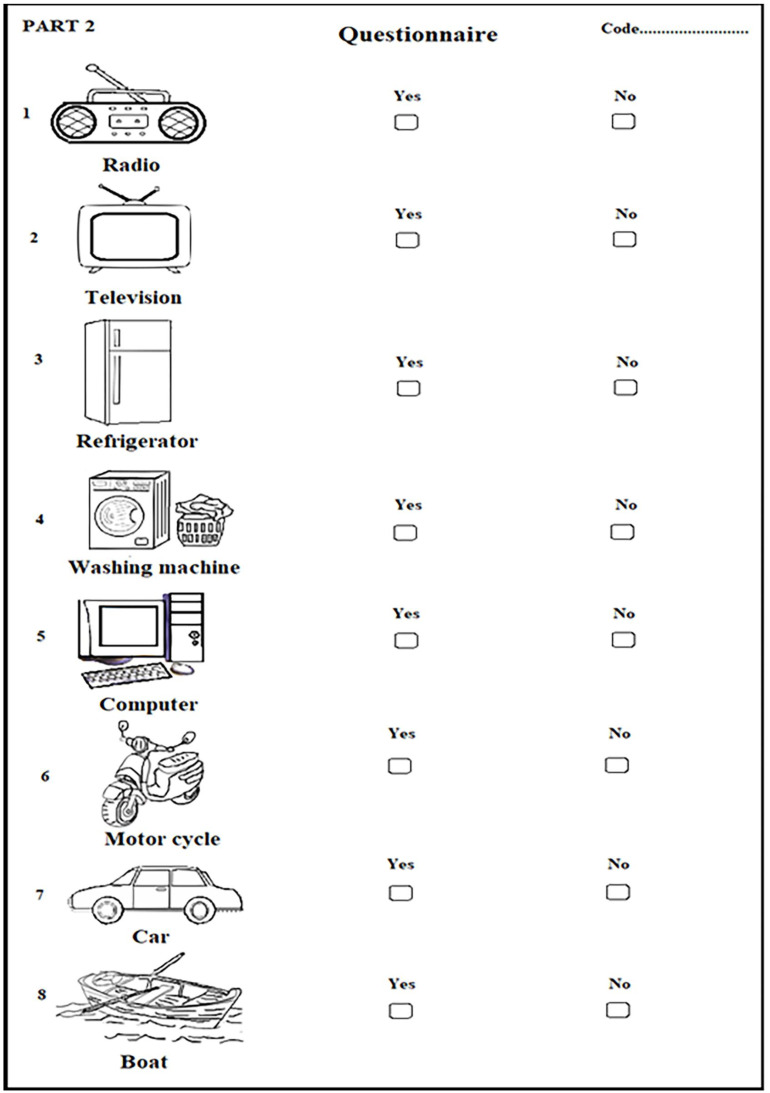
An example of the picture questionnaire used in this study which was used to obtain information on the demographic characteristics, socioeconomic status, and sanitary condition of participants.

The challenge of the present study in the ethnic minority groups living in southwest Thailand is the communication barrier with the participants, some of whom cannot speak the Thai language. To overcome this, we received help from local teenagers enrolled in Thai schools who can speak both the Thai language and the local language to help us communicate with the participants. Furthermore, in finding the factors associated with the prevalence of intestinal parasitic infection, we based our questionnaire on the standard questionnaire from the Ministry of Public Health of Thailand and added some modifications. Instead of the written questionnaire, we used a picture-based questionnaire to gain the accurate data of demographic characteristics, socioeconomic status, and sanitary condition of the participants. The picture questionnaire was designed to collect information on demographic characteristics (age, sex, socioeconomic and sanitary condition, etc.), as shown in Figure 2. The information concerning the socioeconomic status and the sanitary condition of the study population was obtained via personal interviews with the participants using a picture questionnaire.

### 2.4. Statistical Analysis

Data analysis was performed using SPSS Statistics version 18 (SPSS, Inc., Chicago, IL, USA). Demographic data were analyzed using descriptive statistics. Results were expressed as frequency and percentage or mean ± standard deviation. Associations between demographic, anthropometric, and clinical factors and parasitic infection were assessed using a Chi-square test or Fisher’s exact test. A *p*-value less than 0.05 was considered statistically significant.

## 3. Results

### 3.1. Overall Prevalence of Intestinal Parasitic Infection

Of the 780 participants, 691 participants submitted stool samples. The highest prevalence of intestinal parasitic infections was found in the 11–20-year-old age group, followed by those in the 1–10-year-old age group.

Table 1 and Table 2 reveal the overall prevalence of intestinal parasitic infection. The Moken community from Ranong had the highest prevalence of IPIs of 61.8% (95% CI: 56.7–67.1). This further divided into IPIs prevalence of 65% (110/169), 74.4% (58/78), and 43.2% (35/81) in the Moken residing on Chang, Payam, and Lao islands, respectively. The Moken community from Phang Nga had a prevalence of IPIs of 25% (95% CI: 15.7–34.3), and the Orang Laut community from Satun 12.9% (95% CI, 8.9–16.8). A statistically significant difference in IPIs among the three communities was observed (*p* < 0.0001).

### 3.2. Type and Number of Parasitic Infections in Each Study Group

The results revealed that 62% of the study population were infected with one or more types of intestinal parasites. As shown in Table 1, 13.7%, 0.9%, and 11.3% of the Moken community from Ranong were infected with multiple species of STHs, protozoa, and combined STHs and protozoa, respectively, whereas 2.4%, 1.2%, and 2.4% of the Moken from Phang Nga were infected with multiple species of STHs alone, protozoa alone, as well as combined STHs and protozoa, respectively. Only 0.7% of the Orang Laut from Satun were infected with multiple species of protozoa alone and 0.7% of them were infected with combined STHs and protozoa. There was a statistically significant difference in the multiple infection of STHs among the three study groups (*p* < 0.001) but, there was no statistically significant difference in combined STHs and protozoa infection among the three study groups (*p* ≥ 0.55) (Table 1 and Table 2).

### 3.3. Association between the Study Group and Mode of Transmission

Parasites found in the infected individuals were classified by mode of transmission: soil-transmitted helminths (STHs) or food/water-borne protozoa. *Trichuris trichiura*, *Ascaris lumbricoides*, Hookworm, and *Strongyloides stercoralis* were classified as STHs, while *Blastocystis hominis*, *Giardia lamblia*, *Endolimax nana*, *Entamoeba coli*, and *E. histolytica* were classified as food/water-borne protozoa [5,12]. For STH infection status, there was a statistically significant difference between the three study groups (*p* < 0.0001). Moken residing in Ranong had the highest percentage (50.9% and 20.9%) of both STHs and food/water-borne protozoa infection. Approximately 17.9% and 9.5% of the parasites found in the Moken of Phang Nga were STHs and food/water-borne protozoa, respectively. Orang Laut residing in Lipe, Satun, had 2.2% of STHs and 11.5% of food/water-borne protozoa.

Both STHs and food/water-borne protozoa infections were most prevalent in the Moken living in Ranong, whereas the Orang Laut living on Lipe island, Satun, show predominant prevalence (11.5%) of food/water-borne protozoa but very low prevalence (2.2%) of STHs. Regarding food/water-borne protozoa infection, there were statistically significant differences between Moken from Ranong and Phang Nga (*p* = 0.0167), and Moken from Ranong and Orang Laut from Satun (*p* = 0.0019). However, there was no statistically significant difference in water/food-borne protozoa infection between Moken from Phang Nga and Orang Laut from Satun (*p* = 0.6086).

### 3.4. Socioeconomic and Sanitary Conditions

**Figure 3:**
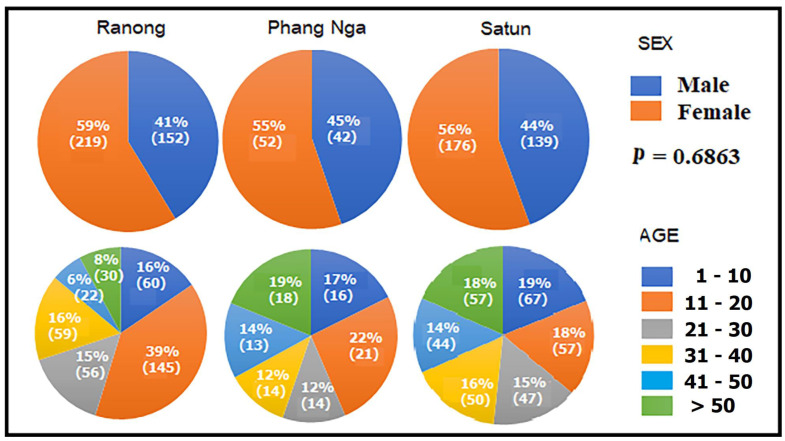
Pie graph reveals sex and age range of the study populations, i.e., the Moken from Ranong, the Moken from Phang Nga, and the Orang Laut from Satun provinces. Parentheses ( ) represent the number of participants.

**Figure 4:**
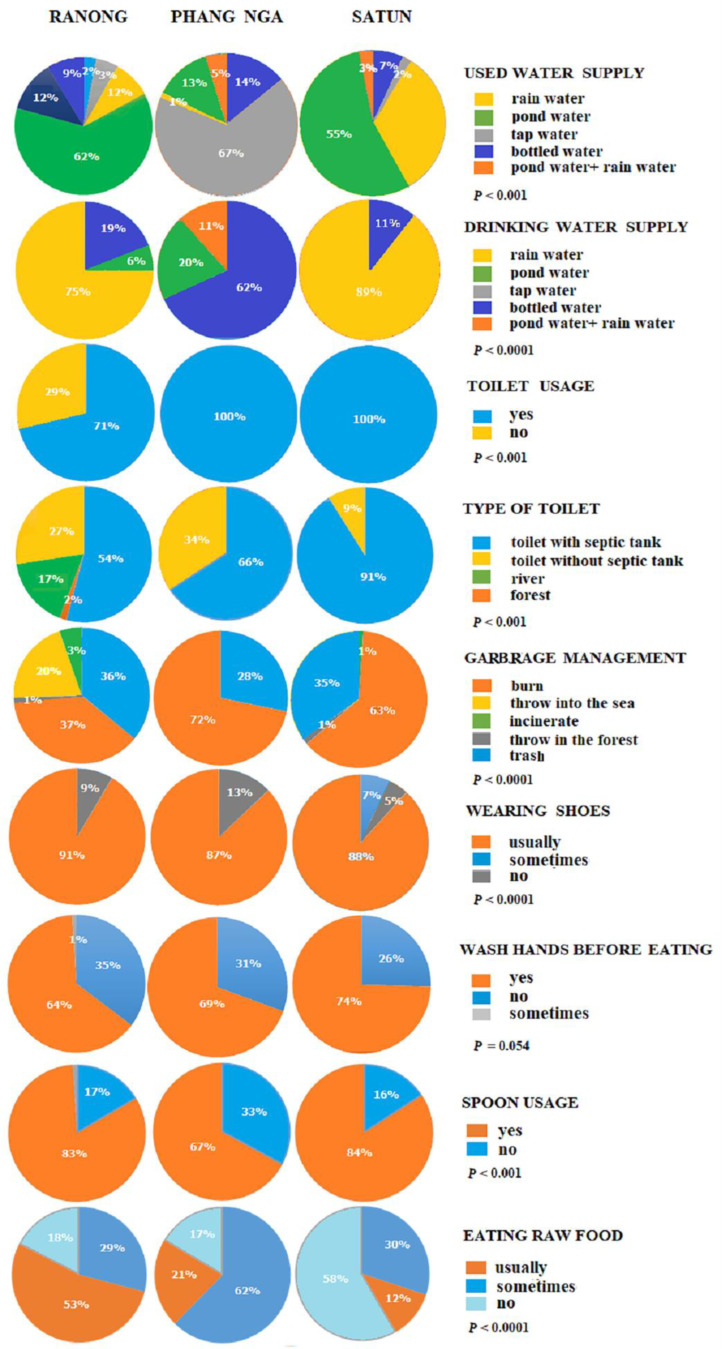
Pie graphs showing data on the socioeconomic status of the study population, i.e., the Moken from Ranong, the Moken from Phang Nga, and the Orang Laut from Satun provinces.

**Figure 5:**
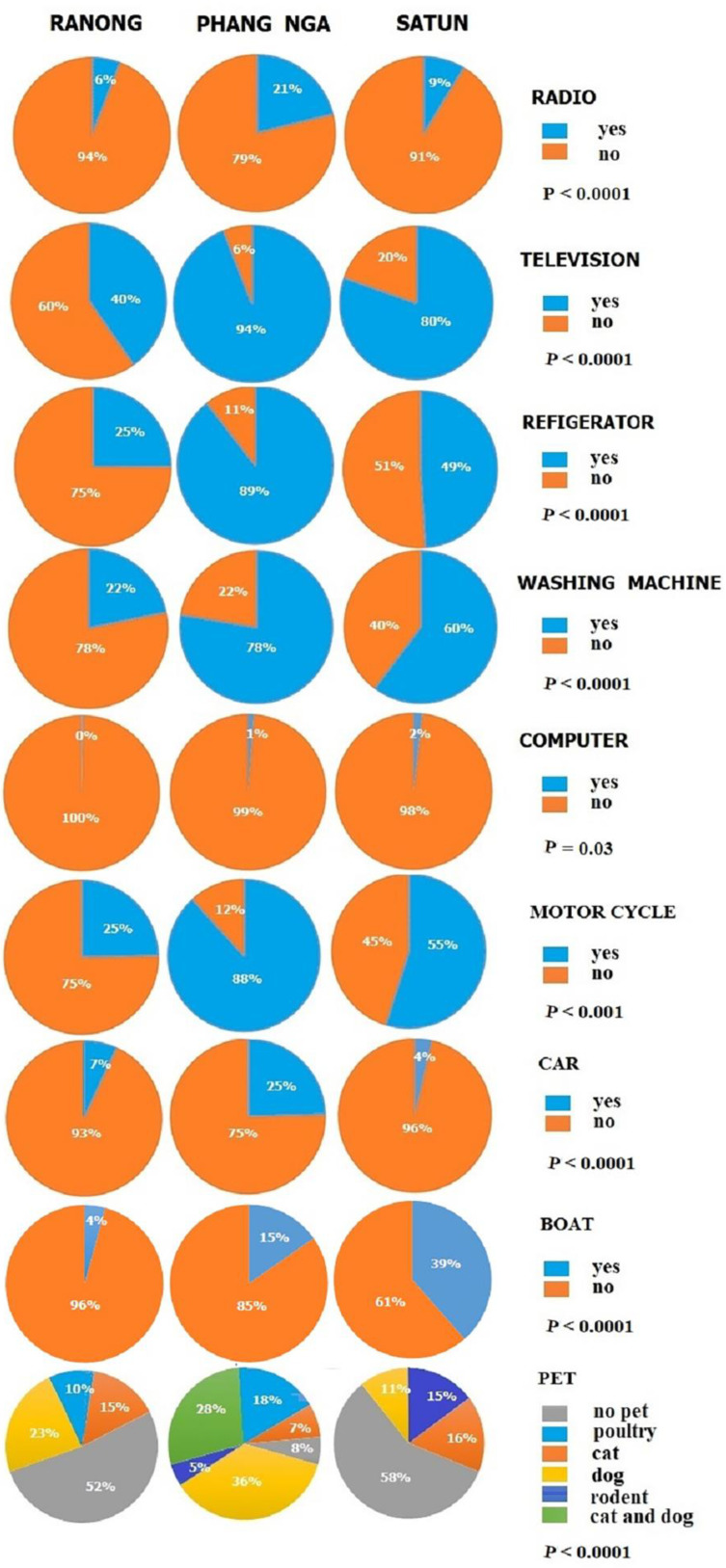
Pie graphs showing data on the sanitary conditions of the study population, i.e., the Moken from Ranong, the Moken from Phang Nga, and the Orang Laut from Satun provinces.

A total of 780 participants, consisting of 339 (43.4%) men and 441 (56.56%) women, were enrolled in the study. This includes 152 (41%) men and 219 (59%) women from the Moken community on the Chang, Pyam, and Lao islands of Ranong province; 42 (45%) men and 52 (55%) women from the Moken community in Ban Thub Tawan, Phang Nga province; and 139 (44%) men and 176 (56%) women from the Orang Laut community on Lipe island, Satun province. The age of the participants ranged from 6 to above 50 years. The age group distribution showed that most of the participants were between 11–20 years old. As shown in Figure 3, the gender of participants shows no statistically significant difference (*p* = 0.6863). Figure 4 and Figure 5 reveal the data regarding the socioeconomic status and sanitary condition of the study population. There was a significant difference in all details of socioeconomic status (*p* < 0.001), except for the possession of a computer which showed no significant difference (*p* = 0.03). For sanitation conditions, there were significant differences in all issues of sanitation conditions (*p* < 0.001), except for washing hands before eating which showed no significant difference (*p* = 0.054). Among the three study groups, Moken living in Phang Nga and Orang Laut living in Satun have better socioeconomic statuses and sanitary conditions than Moken living in Ranong.

## 4. Discussion

In the present study, both direct wet smear and formalin-ethyl acetate concentrations were performed for the detection of intestinal parasites in stool samples of the study population. All parasite-negative samples obtained from direct wet smear method were further subjected to a formalin-ethyl acetate concentration. In the formalin ethyl acetate concentration method, 2 g of stool sample was compared to 2 mg of stool samples used in direct wet smear, increasing the opportunity for intestinal parasitic detection in infected subjects with low worm burden. For detection of protozoa trophozoites and cysts in stool samples, two to three samples collected two to three days apart are necessary as pathogens are shed intermittently [37]. This may be a limitation of the present study since one-time stool samples were collected from the study participants.

There are statistically significant differences in the prevalence of overall IPIs among the Moken living on islands in Ranong, the Moken in Ban Thup Tawan, and the coastal villages of Phan Nga, and the Orang Laut on Lipe island, Satun. The Moken on the islands of Ranong showed highest prevalence of IPIs, including both STHs and protozoa infections, whereas the Orang Laut on Lipe island, Satun, showed the lowest prevalence of STHs infection. The high prevalence of IPIs is closely related to poverty, poor environmental hygiene, and the underdeveloped healthcare service [24,25,38]. This suggested that hygiene, sanitation, and environmental health varied greatly among the three study groups in this study.

Poor sanitation, such as a shortage of drinkable water and a lack of toilets, is a major contributor to intestinal parasite transmission [39,40]. The socioeconomic status and sanitary condition data revealed that the main problem for the Moken in Ranong was the lack of toilets. Only 71% of the villagers had toilets, and among them only 54% of the toilets had a safety tank installed. Approximately 20% of the villagers still defecate into the river or in the forest. In contrast, 100% of the Moken in Phang Nga and the Orang Laut in Satun have toilets.

In addition to the extreme poverty as well as social and health care marginalization, most Moken are stateless, depriving them of access to other citizenship rights, including medical care, education, and employment opportunities [41]. Yet, the data obtained from our interview revealed that the Moken in Phang Nga have a better socioeconomic status than the Moken living in Ranong (*p* < 0.0001).

When the tsunami struck Phang Nga, Thailand, on 26 December 2004, the Moken in Ban Thub Tawan suffered severely from the devastation of housing and fishing boats [41]. After the tsunami, the Moken communities in Ban Thub Tawan were rehabilitated by the Save Andaman Network (SAN), a registered NGO. Many occupational groups were set up to support the affected villagers. SAN also financed the villages to raise pigs and poultry to reduce households’ consumption costs as well as to generate incomes for families. The villagers were trained on handicrafts and woodcrafts as their alternative occupations, and youths in Ban Thup Tawan have gained opportunities to learn and develop their skills through group working and income-generating activities, leading to a better socioeconomic status for the villagers [42,43].

In the year 2010, we performed a survey for IPIs in 117 Orang Laut residing in Rawai village and Ban Tukae village in Phuket province, southern Thailand. A very high prevalence of IPIs (58%) was reported from our previous study (unpublished data). We concluded from our previous survey that four statistically significant factors associated with a high prevalence of IPIs were toilet accessibility, knowledge of hand washing before cooking or eating, and hand washing after using toilets. The main problem of the Orang Laut residing in Rawai village and Ban Tukae village in Phuket was the lack of toilets due to their low socioeconomic status. Households did not have toilets (with or without safety tanks), thus, the villagers defecated on the soil near their houses by the seaside. The seawater spreads eggs or larvae of the parasites when the waves wash away feces, contaminating the area. The significant difference in the IPIs prevalence between the current study and the previous study is because the Orang Laut residing in Phuket are impoverished compared with the Orang Laut residing in Lipe island, Phang Nga.

Lipe is a small island in the Satun province of southwest Thailand, which was originally settled by a group of Malayic-speaking people known as the ‘Orang Laut’ people [44]. The Lipe island economy is largely centered around tourism, especially because of its white sand beaches and famous scuba diving spots. Development on the island is rapidly growing to meet the increase in tourism. In turn, the development of tourism in Lipe island has driven the economy of the local area and enhanced the living standards of the Orang Laut. An increasing number of new generations of Orang Laut are pursuing higher education in colleges or university and can speak fluent Thai. The younger generations here have almost no difficulty adapting to modernization, and electricity and internet access are common. Lipe island has a health promotional hospital and village health volunteers (VHVs). VHVs are villagers who volunteer to work as community health workers in the rural communities of Thailand. They provide health education to the community. They are members of a Thai healthcare alliance, a branch of the ministry of public health established to promote healthcare service communication and collaboration at the primary level and function as key providers of the information regarding health promotion, disease control, and the basic health services available to villagers [30]. Thus, community engagement is a crucial factor for controlling and preventing infectious diseases, including parasitic infection, and maintaining the general health of the community.

In our previous investigation on the prevalence and health effects of intestinal parasitic infection in Thai children living on the mainland of Satun, we found that approximately 18% of the children in the study were infected with at least one parasite, and most children in the previous study were infected with protozoa (12.1%), while 5.5% were found to be infected with helminths [33]. In the present study, a 12.9% prevalence of IPIs (11.5% food/water-borne protozoa and 2.2% STHs) was reported in the Orang Laut on Lipe island, Satun. The concordance prevalence of IPIs of the Orang Laut living on Lipe island and the Thai children living in the mainland of Satun province may be due to the good socioeconomic status of both study groups and may reflect an adequate and functioning health strategy by the provincial public health of Satun. Furthermore, it is proven that there are no associations between ethnicity/race, geographic features, and the prevalence of IPIs.

We also observed that the prevalence, types of parasites, and mode of transmission varied among the study groups. This suggested that hygiene, sanitation, and environment varied greatly among the three study groups evaluated in this study.

The results found that the Moken community from Ranong showed the highest prevalence (both single and multiple infection) of STHs and food/water-borne protozoa followed by the Moken living in Phang Nga. Thus, both STHs and food/water-borne protozoa infection were the main cause of IPIs found in the Moken living on the islands in Ranong and the Moken living in Phang Nga, whereas food/water-borne protozoa were the main cause IPIs in the Orang Laut on Lipe island, Satun.

Contributing factors for the transmission of STHs could be consuming unwashed raw vegetables or fruits contaminated with infective parasites, reluctance to wash hands before meals and after using the toilet, accessibility of latrines, eating raw meat, and dirty fingernails.

Overcrowded households, lack of toilets with or without septic tanks, poor personal hygiene (such as not wearing shoes outdoors, not washing hands before eating, and not using a spoon to eat), as well as the habit of eating unwashed vegetables or fruit should be suspected as the possible causes in areas with a high prevalence of STH infections. Important preventative measures include training the community to wash their hands regularly even without soap, to wash fruits and vegetables thoroughly, and to cook meats and other food dishes well over fire in order to kill any existing helminthic parasites. For food- and water-borne protozoa, the mode of transmission is via contaminated food and water; therefore, problems with contaminated water supply should be suspected as the primary cause of infection. Both the Moken on the island of Ranong and the Orang Laut on Lipe island receive their water supply from a rainwater reservoir and pond. Hence, modernization of the water supply infrastructure and the sewage systems on the island should be funded to prevent contamination of water sources. Additionally, proper water treatment methods, such as boiling of water and water filtration, should be taught to the residents of the island, providing them with proper knowledge concerning safe methods to prepare drinking water.

As seafarers living along the coastlines, seafood is a main staple in daily consumption. Since 50% of the study participants have habits of eating raw food, they might have been infected with anisakis, a nematode that is diagnosed via endoscopy and radiography. Furthermore, more than half of the study participants own cats and dogs, leaving them prone to zoonotic parasitic infection.

A high prevalence of IPIs in humans is positively correlated with poverty, lack of clean water, contamination of the environment by human excreta and animal wastes, and poor personal hygiene and living conditions [45,46]. Our studies have proven that parasitic infection is not directly related to ethnic or geographic features, but the severity of IPIs is mainly due to a low socioeconomic status. Socioeconomic factors, such as a lack of adequate water resources, poor sanitation, and poor hygiene practices, as well as a lack of decent water and sanitation infrastructures [47,48] have repeatedly been proven to correlate with a high prevalence of STHs within a community [49]. Thus, poverty management is the main key success factor of sustainable elimination of IPIs.

The global effort to reduce poverty and improve access to sanitation (UN sustainable development goals (SDG) number one (no poverty) and six (clean water and sanitation)) [49] is expected to have an important impact on the transmission of STHs, further reducing STH prevalence and associated morbidity. Long-term STH control and eventual elimination requires improvements to water, sanitation, and hygiene (WASH) access and practices [50,51]. However, in the absence of any substantial improvement in sanitation, WHO suggests periodic administration of anthelmintic drugs to the affected areas as one of the components of a control strategy to eliminate these infections, along with a progressive reduction in the frequency of mass drug treatment coupled with a regular collection of epidemiologic data to identify any possible prevalence rebound [52]. Adam and co-authors suggested that effective coverage of mass drug administration (MDA) in diverse urban populations can be accomplished via integration of mapping studies, research that identifies context-specific methods to increase MDA coverage, and rigorous monitoring and evaluation [1]. Thus, a customized control strategy, including periodic administration of anthelmintic drugs with a regular collection of epidemiologic data to identify any possible prevalence rebound as well as hygiene and sanitary conditions, should be produced and integrated into the communities of the Orang Laut and the Moken.

## 5. Conclusions

We reached several conclusions from this research. (1) This was the first community-based epidemiological study conducted on IPIs status, which provides baseline epidemiological data on IPIs status and its associated factors among the Moken and the Orang Laut, the minority groups living in coastal areas in southwest Thailand. (2) The advantages of the picture questionnaire are that it is easy to understand and sends a clear message with simple drawings, allowing comprehension even for those of low or no education. (3) Our study found no direct association between parasitic infection and ethnic/geographic features; however, socioeconomic status is the key factor associated with prevalence of intestinal parasitic infection with the observation that the higher prevalence of IPIs is due to the low socioeconomic status, consequently leading to poor hygiene and sanitation practices. (4) Data pertaining to the species of the infected parasites and the mode of transmission from this study may assist in the identification of group-specific vulnerabilities and shortcomings that can be utilized in education to reduce the prevalence of infection in the study areas. (5) The government should increase funds to subsidize health and livelihood improvement to bridge the remaining gap between healthcare access and these communities.

## Figures and Tables

**Table 1:** Prevalence of IPIs in Moken and Orang Laut living in three provinces in Thailand with single and multiple infection.

Province	Ethnic	Geographical Features	Total Subject	IPIS Infected Subject	Helminth	Protozoa	Mix Infection		
							Multiple Species of STHs	Multiple Species of Protozoa	Multiple Species of STHs and Protozoa
Ranong	Moken	Island	328 (100%)	203 (61.8%)	190 (57.9%)	50 (20.9%)	45 (13.7%)	3 (0.9%)	37 (11.3%)
Phang Nga	Moken	Coastal	84 (100%)	21 (25%)	15 (17.9%)	8 (9.5%)	2 (2.4%)	1 (1.2%)	2 (2.4%)
Satun	Orang Laut	Island	279 (100%)	36 (12.9%)	6 (2.2%)	32 (11.5%)	0 (0%)	2 (0.7%)	2 (0.7%)
			**691** **(100%)**	**260** **(37.6%)**	**211** **(30.5%)**	**90** **(13%)**	**47** **(6.8%)**	**6** **(0.9%)**	**41** **(5.9%)**

**Table 2:** Prevalence, types, and species of parasitic infection (helminths and protozoa) found in Moken and Orang Laut living in three provinces in Thailand.

Intestinal Parasitic Infections (IPIs)	Ranong		Phang Nga		Satun	
	Chang, Payam, Lao Islands		Thub Tawan Village		Lipe Island	
	Number (n = 328)	Prevalence (%)	Number (n = 84)	Prevalence (%)	Number (n = 279)	Prevalence (%)
**Overall any parasitic infection**	**203**	**62**	**21**	**25**	**36**	**12.9**
Single-parasitic infection	115	35	16	19	32	11.5
Mix-parasitic infection	88	26.8	5	5.9	4	1.4
**Overall any helminthic infection**	**190**	**57.9**	**15**	**17.9**	**6**	**2.2**
**Overall any protozoa infection**	**50**	**20.9**	**8**	**9.5**	**32**	**11.5**
**STHs**						
*Ascaris lumbricoides*	159	48.5	3	3.6	2	0.7
Hookworm	7	2.1	3	3.6	1	0.4
*Trichuris trichiura*	91	27.7	9	10.7	2	0.7
*Strongyloides stercoralis*	4	1.2	0	0	2	0.7
**Water/food-borne protozoa**						
*Giardia duodenalis*	11	3.4	4	4.8	13	4.7
*Entamoeba histolytica*	4	1.2	0	0	0	0
*Entamoeba coli*	12	3.7	1	1.2	3	1.1
*Endolimax nana*	4	1.2	0	0	0	0
*Blastocystis hominis*	30	9.1	5	6	19	6.8

## Data Availability

Not applicable.
